# Cone-Beam CT-Based Analysis of Temporomandibular Joint Osseous Changes in Orthognathic Surgery Patients: A Retrospective Cross-Sectional Study

**DOI:** 10.3390/diagnostics16010101

**Published:** 2025-12-28

**Authors:** Merve Berika Kadıoğlu, Mehmet Emre Yurttutan, Mehmet Alp Eriş, Meyra Durmaz

**Affiliations:** 1 Department of Orthodontics, Faculty of Dentistry, Ankara University, Ankara 06560, Türkiye; mkadioglu@ankara.edu.tr (M.B.K.); dtmeyradurmaz@gmail.com (M.D.); 2 Department of Oral and Maxillofacial Surgery, Faculty of Dentistry, Ankara University, Ankara 06560, Türkiye; mehmet.alp.eris@gmail.com

**Keywords:** condylar morphology, cone-beam computed tomography, dentofacial deformities, digital imaging techniques, skeletal malocclusion, temporomandibular joint

## Abstract

**Background/Objectives**: The aim of this study is to evaluate pretreatment osseous changes in the temporomandibular joint (TMJ) in orthognathic surgery patients using cone-beam computed tomography (CBCT) and to determine the distribution of the findings according to sagittal skeletal malocclusion groups. **Methods**: CBCT images of 103 patients (206 condyles) were retrospectively analyzed. Patients were classified as Class I, II, and III based on ANB angles. Condylar morphology was assessed for flattening, sclerosis, erosion, osteophyte formation, and subchondral bone cysts. All evaluations were performed by a single investigator (κ = 0.87). Group differences were analyzed using the chi-square test with Bonferroni correction (*p* < 0.05). **Results**: The most frequent alteration was flattening (29.6%), followed by sclerosis (11.2%), erosion (10.7%), osteophyte formation (8.3%), and subchondral bone cysts (4.4%). No significant sex-related differences were found (*p* > 0.05). A significant difference was observed only in sclerosis (*p* = 0.049), which was more prevalent in Class I than Class III. Flattening predominated in all groups, while erosion and osteophytes were more common in Class II, and sclerosis was more frequent in Class I. **Conclusions**: This study demonstrated that condylar flattening was the most common morphological alteration in orthognathic patients across all skeletal malocclusion groups. The higher prevalence of sclerosis in Class I compared with Class III suggests that mandibular positioning may influence adaptive and degenerative remodeling processes of the TMJ. This study emphasizes the importance of CBCT evaluation for detecting osseous changes in TMJ before orthognathic surgery and demonstrates that pre-existing alterations may impact surgical stability and postoperative functional outcomes.

## 1. Introduction

The temporomandibular joint (TMJ) is a complex structure located between the mandibular condyle and the glenoid fossa of the temporal bone, which undergoes continuous physiological remodeling. Facilitating essential functions such as mastication, speech, and swallowing, the TMJ also plays a crucial role in maintaining the functional integrity of the craniofacial complex [1,2].

Temporomandibular disorders (TMDs) affect the masticatory muscles, the TMJ, and associated structures. With a multifactorial etiology, these disorders arise from the interaction of mechanical and biological factors and may, over time, alter the morphology of the joint’s hard tissues (the condyle and the articular fossa/eminence) [3]. In particular, skeletal malocclusions and variations in craniofacial morphology can adversely affect the distribution of loads transmitted to the joint, reducing its capacity to tolerate these forces and thereby predisposing it to degenerative changes. Degenerative changes, also defined as osteoarthritis, represent a chronic and progressive condition characterized by structural deterioration of the bone surface. Radiographic manifestations typically include condylar flattening, osteophyte formation, subchondral bone cysts, sclerosis, and erosion [4,5].

The morphological and functional status of the TMJ can directly influence the outcomes of surgical planning; pre-existing condylar alterations may increase the risk of postoperative joint dysfunction, instability, and relapse [6]. Therefore, a comprehensive evaluation of the TMJ in patients scheduled for orthognathic surgery is of critical importance, both for treatment planning and for ensuring the predictability of postoperative outcomes.

Conventional radiographic techniques are inherently limited in evaluating anatomical changes across three spatial planes because of factors such as magnification errors, structural superimposition, and projection-related distortions. In contrast, cone-beam computed tomography (CBCT) has emerged as a reliable imaging modality for three-dimensional assessment of the craniofacial complex, providing accurate visualization of osseous structures. Owing to its high spatial resolution and geometric accuracy, CBCT is particularly well suited for detailed evaluation of dentofacial deformities and TMJ morphology, especially in the analysis of hard tissue and bony components [7,8].

The question of whether variations in TMJ structure are associated with specific types of dentofacial deformities has long been a subject of scientific debate, and it has frequently been reported that differences exist in the size or shape of the skeletal components of the TMJ among various types of malocclusions [9,10,11].

In the literature, several studies have investigated the distribution of temporomandibular morphology according to vertical facial patterns and malocclusions [9,12]. However, the evaluation of pre-treatment CBCT records of patients who have undergone orthognathic surgery provides an opportunity to expand these findings and offer a more comprehensive perspective.

The aim of this study is to assess the skeletal morphology of the TMJ in orthognathic surgery patients using CBCT images obtained prior to orthodontic treatment and surgery and to determine the distribution of the findings according to different types of skeletal malocclusion.

## 2. Materials and Methods

### 2.1. Study Design and Ethical Approval

The study was approved by the Ethics Committee of the Faculty of Dentistry, Ankara University (Decision Date: 6 October 2025, Decision No: 14/1). This retrospective study was conducted based on the records of 124 patients who were planned to undergo orthognathic surgery between 2020 and 2024 at the Departments of Orthodontics and Oral and Maxillofacial Surgery, Faculty of Dentistry, Ankara University. Patient records available at the Faculty of Dentistry, Ankara University, were reviewed. Informed consent forms routinely obtained from all participants prior to surgery included permission for the use of their data after the treatment. This retrospective cross-sectional study was conducted in accordance with the STROBE (Strengthening the Reporting of Observational Studies in Epidemiology) guidelines.

During the preparation of this manuscript/study, the author(s) used ChatGPT 5.0 and Rubriq for the purposes of translation, improving the English quality, and language editing. The authors have reviewed and edited the output and take full responsibility for the content of this publication.

### 2.2. Participants

The sample size of the study was determined using the G*Power 3.1 software. An a priori power analysis for a one-way ANOVA test, assuming a medium-to-high effect size (f = 0.40), an alpha level of 0.05, and a statistical power of 80%, indicated that a minimum of 22 participants per group—resulting in a total of 66 participants—would be required for adequate statistical validity.

**Figure 1:**
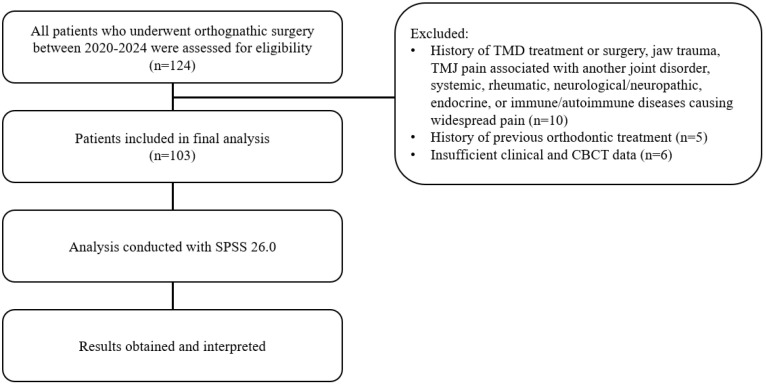
Flowchart of the study.

Patients with a history of TMD treatment or surgery, jaw trauma, TMJ pain associated with another joint disorder, systemic, rheumatic, neurological/neuropathic, endocrine, or immune/autoimmune diseases causing widespread pain (*n* = 10), a history of previous orthodontic treatment (*n* = 5), or insufficient clinical and CBCT data (*n* = 6) were excluded from the study (Figure 1).

A total of 103 individuals and 206 condyles were included in the evaluation. Patients were divided into three groups based on sagittal skeletal classification: Class I (*n* = 36; 11 males, 25 females), Class II (*n* = 30; 7 males, 23 females), and Class III (*n* = 37; 18 males, 19 females). Consecutive CBCT records were retrospectively assessed for sample selection (Table 1).

Sagittal skeletal relationships were analyzed using Dolphin Imaging^®^ software version 11.8 (Chatsworth, CA, USA). Patients were classified into three groups based on the ANB angle, a cephalometric measurement formed between Point A, Nasion, and Point B, which evaluates the relative sagittal position of the maxilla and mandible: Class I (0° ≤ ANB ≤ 4°), Class II (ANB > 4°), and Class III (ANB < 0°). However, since the ANB angle only reflects the relative position of the jaws to each other, the Sella–Nasion–Point B (SNB) angle was also considered to more accurately assess the position of the mandible relative to the cranium. The normal range of the SNB angle was accepted as 78–82°; values below 78° were interpreted as mandibular retrognathia (Class II), values between 78° and 82° as normal mandibular position (Class I), and values above 82° as mandibular prognathia (Class III).

### 2.3. Measurement Instruments

The reconstruction of the mandibular condyle, fossa/articular eminence, and the osseous components of the TMJ was performed based on methodological approaches reported in previous studies [13,14]. The right and left TMJ were evaluated using reconstructed lateral sections obtained perpendicular to the long axis of the condyle, coronal sections parallel to the long axis, and lateral views depicting the center of the joint.

Bilateral TMJ images were evaluated using CBCT scans obtained with a flat-panel-based NewTom 7G device (QR s.r.l., Verona, Italy) following a standard scanning protocol (110 kVp, 29 mA, 0.3 mm isotropic voxel, 0.6 mm slice thickness). All examinations were performed by the same experienced surgeon. To ensure observer reliability, 20 randomly selected CBCT records were re-evaluated by the same investigator after a two-week interval, and the intraobserver agreement kappa coefficient was found to be 0.87.

**Figure 2:**
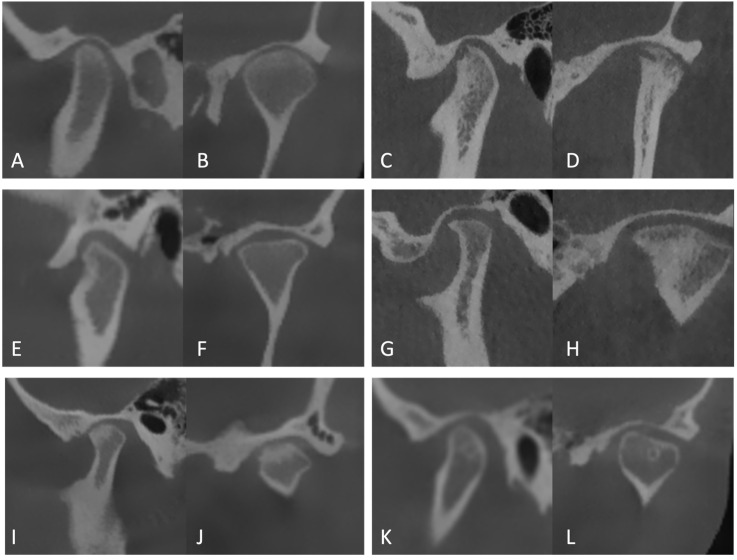
CBCT images of the mandibular condyles demonstrating normal morphology (**A**,**B**), erosion (**C**,**D**), flattening (**E**,**F**), osteophyte (**G**,**H**), sclerosis (**I**,**J**), and subchondral bone cyst (**K**,**L**) in sagittal and coronal views.

An image of a mandibular condyle with a healthy condylar head is presented in Figure 2. The mandibular condyles were evaluated for morphological alterations based on the criteria described by Kiliç et al., including flattening (loss of the rounded contour of the surface), bone sclerosis (thickening of the cortical layer and increased bone density in load-bearing areas), erosion (loss of continuity of the articular cortex), osteophyte formation (proliferation of bone tissue with a sclerotic margin, protruding outward from the articular surface edge with an angular configuration), and subchondral bone cysts (formation of a cavity beneath the articular surface that differs from the normal structure) [5] (Figure 2).

### 2.4. Statistical Analyses

Data analysis was performed using SPSS version 26. Descriptive statistics were initially performed to describe the distribution of independent variables (sex and skeletal malocclusion groups) and dependent variables related to TMJ condylar morphology, including flattening, sclerosis, erosion, osteophyte formation, and subchondral bone cysts.

Chi-square tests were used to compare the prevalence of condylar morphological changes (flattening, sclerosis, erosion, osteophyte formation, and subchondral bone cysts) across skeletal malocclusion groups (Class I, II, and III) and between sexes. When a significant overall difference was detected among skeletal classes, post hoc pairwise comparisons were performed using the Bonferroni correction to identify the specific groups responsible for the difference.

## 3. Results

A total of 206 condyles from 103 patients were evaluated in the study. Of these, 36 were male (35.0%) and 67 were female (65.0%), with female patients being represented at a higher proportion compared to males.

In male patients, the frequency distribution of condylar morphological changes was as follows: flattening (26.4%), erosion (11.1%), sclerosis (11.1%), osteophyte formation (6.9%), and subcortical cysts (2.8%). In female patients, the distribution was flattening (31.3%), sclerosis (11.2%), erosion (10.4%), osteophyte formation (9%), and subcortical cysts (5.2%). Statistical analysis revealed no significant differences between sexes in the prevalence of erosion (*p* = 0.883), flattening (*p* = 0.458), osteophyte formation (*p* = 0.617), sclerosis (*p* = 0.956), or subcortical cysts (*p* = 0.413) (*p* > 0.05) (Table 2).

In the patient-based analysis, no statistically significant differences were observed among skeletal classes in terms of the prevalence of erosion (*p* = 0.337), flattening (*p* = 0.374), osteophyte formation (*p* = 0.469), sclerosis (*p* = 0.190), or subchondral bone cysts (*p* = 0.222) (*p* > 0.05) (Table 3).

In Class I patients, the distribution of condylar morphological changes was as follows: flattening (37.5%), sclerosis (18.1%), erosion (8.3%), osteophyte formation (8.3%), and subcortical cysts (4.2%). In Class II patients, the distribution by frequency was flattening (31.7%), erosion (13.3%), osteophyte formation (13.3%), sclerosis (10%), and subcortical cysts (6.7%). In the Class III group, flattening was most frequently observed (20.3%), followed by erosion (10.8%), sclerosis (5.4%), osteophyte formation (4.1%), and subcortical cysts (2.7%). Statistical analysis revealed a significant difference among the groups only in the prevalence of sclerosis (*p* = 0.049; *p* < 0.05). Post hoc comparisons with Bonferroni correction indicated that the presence of sclerosis was higher in Class I patients compared to Class III. No significant differences were observed among the groups for erosion (*p* = 0.651), flattening (*p* = 0.068), osteophyte formation (*p* = 0.152), or subcortical cysts (*p* = 0.533) (*p* > 0.05) (Table 4).

Among the morphological changes observed in all condyles, flattening was the most prevalent finding (29.6%), followed by sclerosis (11.2%), erosion (10.7%), osteophyte formation (8.3%), and subchondral bone cysts (4.4%) (Table 5).

## 4. Discussion

The TMJ, together with its associated bony structures, muscles, and articular disk, undergoes continuous remodeling and is considered one of the most complex functional units of the human body. Ongoing physiological remodeling in the TMJ maintains biomechanical harmony between the articular surfaces, contributing both to the preservation of joint integrity and to the continuity of an optimal occlusal relationship within the dentoalveolar system. However, when stress factors exceeding the adaptive capacity of the joint are introduced—such as posteriorly directed forces on the condyle in mandibular retrognathia or excessive functional loading in mandibular prognathism—the physiological remodeling process may be disrupted, and adaptive responses may lead to pathological changes. This can manifest as morphological alterations, volumetric reductions, or dimensional irregularities in the condyle, resulting in dysfunctional remodeling [15,16,17].

Understanding the adaptive and degenerative mechanisms of the TMJ in the context of skeletal discrepancies remains a subject of ongoing debate in the literature.

This study provides important insights into the evaluation of osteoarthritic changes in TMJ morphology among individuals requiring orthognathic surgery, based on CBCT imaging. In the TMJ, osteoarthritis is not a discrete pathological entity but rather represents a continuum of degenerative and adaptive remodeling processes, encompassing radiographic findings such as flattening, sclerosis, erosion, osteophyte formation, and subchondral bone cysts. The findings demonstrated that condylar flattening was the most prevalent alteration across all skeletal malocclusion groups, followed by sclerosis, erosion, and osteophyte formation, whereas subchondral bone cysts were the least frequently observed. Furthermore, the higher prevalence of sclerosis in individuals with Class I malocclusion suggests that mandibular positioning may play a decisive role in the adaptive and degenerative processes affecting the TMJ. These results underscore the necessity of considering joint morphology in orthognathic surgery planning.

In the present study, condylar morphological changes were additionally evaluated using a patient-based approach, in which unilateral and bilateral findings were combined to reflect the overall joint status. This approach was adopted to account for shared etiological and biomechanical factors affecting both temporomandibular joints and is considered clinically more meaningful than side-based evaluations. However, patient-based analysis did not reveal statistically significant differences in the distribution of condylar morphological changes among skeletal malocclusion groups, suggesting that these alterations may be influenced by multifactorial mechanisms beyond sagittal skeletal classification alone.

In the literature, degenerative changes in condylar morphology have been reported to occur more frequently in female patients [5,18,19]. It has been emphasized that the primary reason for this susceptibility in women is hormonal differences. In particular, estrogen deficiency and decreases in hormone levels may increase the risk of condylar resorption [20]. In the present study, consistent with the literature, degenerative changes were observed more frequently in female patients (65.0%).

In the literature, several studies have evaluated the relationship between age and TMJ morphological changes. Cruzoé-Rebello et al. and Isberg et al. reported no significant association between increasing age and condylar bone changes; however, they noted that TMJ alterations were more frequently observed in individuals aged 20–49 years [21,22]. In the present study, the mean age of the evaluated patients was 27.8 years, and the sample was kept homogeneous to minimize age-related differences.

Various radiological findings observed in the TMJ reflect different stages of the osteoarthritic process. Early changes are typically characterized by erosive lesions, whereas osteophyte formation is considered a late finding that represents reparative mechanisms occurring on the bone surface. Flattening and sclerosis, on the other hand, are generally regarded as reflections of the joint’s physiological adaptive response [23,24].

Several studies have evaluated changes in TMJ morphology in individuals with skeletal Class I malocclusion. Walewski et al. reported flattening and osteophyte formation as the most frequently observed condylar changes, whereas Krisjane et al. indicated that flattening and sclerosis were predominant [25,26]. In the present study, among Class I malocclusion patients with detected condylar morphological changes, flattening was the most frequently observed finding, followed by sclerosis. These results are largely consistent with those reported in the literature. Our study also revealed that individuals with Class I malocclusion exhibited a higher prevalence of flattening and sclerosis compared to Class II and Class III malocclusion groups. This finding suggests that anterior or posterior positioning of the mandible in the sagittal plane may trigger dysfunctional remodeling processes within the osteoarthritic spectrum of the TMJ, particularly manifesting as chronic adaptive changes such as condylar flattening and sclerosis. The results obtained are consistent with those reported by Krisjane et al. [26].

The normal position of the mandible in the sagittal plane and Class I malocclusion may trigger adaptive remodeling processes such as flattening and sclerosis by imposing more balanced but chronic compressive loads on the condyle; this explains the biomechanical differences observed in load directions and intensities in different types of malocclusions [27]. Furthermore, these findings emphasize the importance of considering condylar surface adaptations in orthognathic surgical planning and provide radiological clues for evaluating postoperative joint stability.

In the literature, numerous studies have evaluated temporomandibular condylar changes in individuals with skeletal Class II malocclusion associated with mandibular retrognathia. These studies have reported condylar flattening and osteophyte formation as the most frequently observed findings, whereas the presence of subchondral bone cysts has been identified as the least common alteration [25,26,28]. However, some studies have reported different findings. For instance, Oliveira et al., contrary to the general trend in the literature, reported that the most frequently observed pathology in Class II skeletal malocclusion patients with temporomandibular disorder was subchondral bone cysts, followed by the prevalence of erosion and osteophytes [29]. Chen et al. reported that, in female patients with skeletal Class II malocclusion, osteophyte formation and erosion were the most frequently observed findings, followed by flattening and sclerosis, respectively [30]. In the present study, largely consistent with the literature, flattening (31.7%) was the most frequently observed condylar change in individuals with Class II malocclusion associated with mandibular retrognathia. This was followed by erosion (13.3%) and osteophyte formation (13.3%) at equal rates.

Studies conducted on individuals with Class III malocclusion associated with mandibular prognathism have reported the presence of different degenerative changes in the TMJ. Walewski et al. and Tran Duy et al. found that the most frequently observed findings were condylar flattening and erosion [25,31]. Krisjane et al. reported that the most common findings were flattening and osteophyte formation [26]. In the present study, flattening was the most frequently observed alteration in individuals with skeletal Class III malocclusion, followed by erosion.

Consistent with findings in the literature, the least frequently observed condylar change across all malocclusion groups was the presence of subchondral bone cysts [32,33]. The low prevalence of subchondral bone cysts suggests that this finding may not be directly associated with jaw deformities.

Differences in skeletal patterns may alter the distribution of functional orthopedic stresses acting on the TMJ, leading to various adaptive or degenerative changes in the anatomical morphology of the joint [15]. In a study conducted by Schellhas et al., it was reported that individuals with retrognathic mandibles exhibited TMJ irregularities and anterior disk displacement more frequently, and that the severity of degenerative changes in the joint was directly associated with the degree of retrognathia [34]. In the present study, when skeletal malocclusion groups were evaluated separately, flattening was identified as the most frequently observed condylar change across all groups. In the Class II skeletal malocclusion group, flattening was most frequently observed, followed by erosion and osteophyte formation, which can be interpreted as radiographic manifestations of different phases within the progression of TMJ osteoarthritis, with erosion representing an early degenerative phase and osteophyte formation reflecting a later-stage remodeling response. In the Class III skeletal malocclusion group, flattening was followed by erosion, an early-stage finding, and sclerosis, which is considered an adaptive response of the joint. These findings suggest that mandibular retrognathia may predispose the TMJ to progressive phases of osteoarthritis, whereas mandibular prognathism may predominantly be associated with compensatory osteoarthritic remodeling, reflecting a more physiological adaptive phase of the same disease spectrum. In conclusion, consistent with the literature, our results indicate that the direction of functional orthopedic forces transmitted to the joint in cases of mandibular retrognathia may be a determining factor in the development of degenerative responses [12,34,35].

A notable strength of this study is the comprehensive evaluation of osseous changes in the TMJ using high-resolution CBCT, which offers reliable multiplanar imaging with minimal radiation exposure. The stratification of participants by sagittal skeletal pattern, based on both ANB and SNB values, enabled a more refined analysis of mandibular-origin malocclusions. Radiologic evaluations performed by a single, calibrated examiner with high intraobserver agreement further enhanced methodological rigor. Importantly, osteoarthritic changes were interpreted within the context of pathophysiological mechanisms rather than isolated findings. The use of appropriate statistical analyses, ethical approval, and inclusion of orthognathic surgery candidates further substantiate the study’s clinical relevance and validity.

### Limitations

This study only included CBCT-based morphological evaluation, and clinical symptoms were not included in the analysis. Furthermore, the fact that the data was obtained from a single center may limit the generalizability of the results to larger populations. Therefore, it is recommended that these findings be supported by future studies that are multicenter and integrate clinical-functional data.

## 5. Conclusions

This study underscores the clinical relevance of TMJ morphology in orthognathic surgery patients and highlights the influence of sagittal mandibular position on condylar remodeling patterns. Variations in mandibular positioning appear to affect the biomechanical environment of the joint, which may have implications for joint stability and surgical planning. Accordingly, systematic preoperative CBCT evaluation of the TMJ may support more informed surgical decision-making by aiding in the assessment of joint morphology and potential postoperative risks. Future prospective studies incorporating standardized clinical and functional assessments are warranted to further clarify the clinical significance of these radiological findings.

## Figures and Tables

**Table 1:** Age distribution of skeletal malocclusion groups.

Skeletal Malocclusion	*n*	Mean Age	Ratio
Class I	36	28.0 ± 5.2	35.0%
Class II	30	28.1 ± 5.1	29.1%
Class III	37	27.1 ± 7.2	35.9%
Total	103	27.8 ± 5.8	100%

*n*: number of participants.

**Table 2:** Prevalence of condylar morphological changes by sex.

Condylar Change		Male		Female		*p* *
		*n*	%	*n*	%	
Erosion	Absence	64	88.9	120	89.6	0.883
	Presence	8	11.1	14	10.4	
Flattening	Absence	53	73.6	92	68.7	0.458
	Presence	19	26.4	42	31.3	
Osteophyte	Absence	67	93.1	122	91	0.617
	Presence	5	6.9	12	9	
Sclerosis	Absence	64	88.9	119	88.8	0.956
	Presence	8	11.1	15	11.2	
Subchondral Bone Cyst	Absence	70	97.2	127	94.8	0.413
	Presence	2	2.8	7	5.2	

n: number of participants; * Chi-square significance value.

**Table 3:** Distribution of condylar changes according to skeletal malocclusion groups based on patient-level analysis.

Condylar Change		Class I		Class II		Class III		*p* *
		*n*	%	*n*	%	*n*	%	
Erosion	Absence	31	86.1	23	76.7	32	86.5	0.337
	Unilateral	4	11.1	6	20	2	5.4	
	Bilateral	1	2.8	1	3.3	3	8.1	
Flattening	Absence	17	47.2	17	56.7	25	67.6	0.374
	Unilateral	11	30.6	7	23.3	9	24.3	
	Bilateral	8	22.2	6	20	3	8.1	
Osteophyte	Absence	31	86.1	23	76.7	34	91.9	0.469
	Unilateral	4	11.1	6	20	3	8.1	
	Bilateral	1	2.8	1	3.3	0	0	
Sclerosis	Absence	28	77.8	27	90	35	94.6	0.190
	Unilateral	2	5.6	1	3.3	0	0	
	Bilateral	6	16.7	2	6.7	2	5.4	
Subchondral Bone Cyst	Absence	30	83.3	20	66.7	30	8.1	0.222
	Unilateral	6	16.7	10	33.3	7	18.9	

n: number of participants; * Chi-square significance value.

**Table 4:** Prevalence of condylar morphological changes by skeletal malocclusion group.

Condylar Change		Class I		Class II		Class III		*p* *
		*n*	%	*n*	%	*n*	%	
Erosion	Absence	66	91.7	52	86.7	66	89.2	0.651
	Presence	6	8.3	8	13.3	8	10.8	
Flattening	Absence	45	62.5	41	68.3	59	79.7	0.068
	Presence	27	37.5	19	31.7	15	20.3	
Osteophyte	Absence	66	91.7	52	86.7	71	95.9	0.152
	Presence	6	8.3	8	13.3	3	4.1	
Sclerosis	Absence	59	81.9 ^a,b^	54 ^a,b^	90 ^a,b^	70 ^b^	94.6 ^b^	0.049
	Presence	13	18.1 ^a^	6 ^a,b^	10 ^a,b^	4 ^b^	5.4 ^b^	
Subchondral Bone Cyst	Absence	69	95.8	56	93.3	72	97.3	0.533
	Presence	3	4.2	4	6.7	2	2.7	

*n*: number of participants; * Chi-square significance value. ^a,b^ *Statistical significance was evaluated after applying the Bonferroni correction*.

**Table 5:** Prevalence of condylar morphological changes in total condyle count by skeletal malocclusion group.

Condylar Change		Class I		Class II		Class III		*p* *
		*n*	%	*n*	%	*n*	%	
Erosion	Absence	66	32	52	25.2	66	32	0.651
	Presence	6	2.9	8	3.9	8	3.9	
Flattening	Absence	45	21.8	41	19.9	59	28.6	0.068
	Presence	27	13.1	19	9.2	15	7.3	
Osteophyte	Absence	66	32	52	25.2	71	34.5	0.152
	Presence	6	2.9	8	3.9	3	1.5	
Sclerosis	Absence	59 ^a^	28.6	54 ^a^	26.2	70 ^b^	34	0.049
	Presence	13 ^a^	6.3	6 ^a^	2.9	4 ^b^	1.9	
Subchondral Bone Cyst	Absence	69	33.5	56	27.2	72	35	0.533
	Presence	3	1.5	4	1.9	2	1	

*n*: number of participants; * Chi-square significance value. ^a,b^ *Statistical significance was evaluated after applying the Bonferroni correction*.

## Data Availability

The data presented in this study are available on request from the corresponding author due to ethical restrictions.

## Abbreviations

The following abbreviations are used in this manuscript:

TMJ	Temporomandibular Joint
TMD	Temporomandibular Disorders
CBCT	Cone-Beam Computed Tomography
ANB	A Point–Nasion–B Point Angle
SNB	Sella–Nasion–B Point Angle
